# Association of Muscle Strength With All‐Cause Mortality in the Oldest Old: Prospective Cohort Study From 28 Countries

**DOI:** 10.1002/jcsm.13619

**Published:** 2024-10-22

**Authors:** Lars Louis Andersen, Rubén López‐Bueno, Rodrigo Núñez‐Cortés, Eduardo Lusa Cadore, Ana Polo‐López, Joaquín Calatayud

**Affiliations:** ^1^ National Research Centre for the Working Environment Copenhagen Denmark; ^2^ Exercise Intervention for Health Research Group (EXINH‐RG), Department of Physiotherapy University of Valencia Valencia Spain; ^3^ Department of Physical Medicine and Nursing University of Zaragoza Zaragoza Spain; ^4^ Department of Physical Therapy, Faculty of Medicine University of Chile Santiago Chile; ^5^ Exercise Research Laboratory, School of Physical Education, Physiotherapy and Dance Universidade Federal do Rio Grande do Sul Porto Alegre Brazil

**Keywords:** longevity, sarcopenia, frailty, ageing

## Abstract

**Background:**

Ageing is associated with a gradual loss of muscle strength, which in the end may have consequences for survival. Whether muscle strength and mortality risk associate in a gradual or threshold‐specific manner remains unclear. This study investigates the prospective association of muscle strength with all‐cause mortality in the oldest old.

**Methods:**

We included 1890 adults aged ≥ 90 years (61.6% women, mean age 91.0 ± 1.5 years) from 27 European countries and Israel participating in the Survey of Health, Ageing and Retirement in Europe (SHARE) study. Muscle strength was assessed using handgrip dynamometry (unit: kilogram). Using time‐varying Cox regression with restricted cubic splines, we determined the prospective association of muscle strength with mortality, controlling for age, sex, smoking, BMI, marital status, education, geographical region and self‐perceived health.

**Results:**

Over a mean follow‐up of 4.2 ± 2.4 years, more than half of the participants died (*n* = 971, 51.4%). The mean handgrip strength was 20.4 ± 8.0 kg for all participants, with men (26.7 ± 7.5 kg) showing significantly higher strength than women (16.4 ± 5.4 kg) (*p* < 0.001). Using the median level of muscle strength as reference (18 kg), lower and higher levels were associated in a gradual and curvilinear fashion with higher and lower mortality risk, respectively. The 10^th^ percentile of muscle strength (10 kg) showed a hazard ratio (HR) of 1.27 (95% CI 1.13–1.43, *p* < 0.001). The 90^th^ percentile (31 kg) showed an HR of 0.69 (95% CI 0.58–0.82, *p* < 0.001). Stratified for sex, the median levels of muscle strength were 26 kg for men and 16 kg for women. The 10^th^ percentile of muscle strength showed HRs of 1.33 (95% CI 1.10–1.61, *p* < 0.001) at 15 kg for men and 1.19 (95% CI 1.05–1.35, *p* < 0.01) at 10 kg for women. The 90^th^ percentile of muscle strength showed HRs of 0.75 (95% CI 0.59–0.95, *p* < 0.01) at 35 kg for men and 0.75 (95% CI 0.62–0.90, *p* < 0.001) at 23 kg for women. Sensitivity analyses, which excluded individuals who died within the first 2 years of follow‐up, confirmed the main findings.

**Conclusion:**

Rather than a specific threshold, muscle strength is gradually and inversely associated with mortality risk in the oldest old. As muscle strength at all ages is highly adaptive to resistance training, these findings highlight the importance of improving muscle strength in both men and women among the oldest old.

## 1 Introduction

Skeletal muscle comprises about 40% of the body weight and is essential for activities of daily living and overall health [1]. Ageing is associated with a range of physiological changes, including alterations in body composition, musculoskeletal health, cardiovascular function, endocrine systems and inflammatory markers [2, 3]. Musculoskeletal changes include a decline in muscle mass and strength and an increase in intramuscular fat [4]. Muscle strength typically peaks between the ages of 30 and 39 years followed by a gradual decline of about 1%–3% per year [5]. In older people, this decline can have important consequences for activities of daily living such as gait and sit‐to‐stand ability [6, 7]. Skeletal muscle also acts as an endocrine organ important for overall health. Physical activity stimulates skeletal muscles to release myokines, which are involved in body weight regulation, insulin sensitivity and reduced levels of low‐grade inflammation [8]. Thus, skeletal muscles are crucial for overall health.

Handgrip strength provides an easily accessible measure of overall muscle strength that is related to skeletal muscle mass [9, 10]. Previous studies have documented an association of handgrip strength in older adults with a range of diseases and disorders such as hypertension, Parkinson's disease, stroke, work limitations and mortality due to various causes [11, 12, 13, 14, 15, 16]. However, few studies have assessed the importance of handgrip strength on survival in the ‘oldest old’, a fast‐growing population that challenges healthcare systems because of their high prevalence of multimorbidity and polypharmacy [17, 18]. The American Geriatric Society and the World Health Organization define the oldest old as individuals aged 80 years and above, whereas the British Geriatrics Society considers those aged 85 and above as fitting this category. As life expectancy continues to increase, recent studies even consider those aged 90 years or older as the oldest old [19, 20]. A study from the Netherlands found that 85‐year‐olds in the lowest tertile of handgrip strength were at increased risk of dying during follow‐up [21]. A study from Belgium found that high handgrip strength was associated with lower risk of mortality in people aged 80 years or older [22]. Likewise, a study with 81 participants aged 90 years or older from Brazil found that a higher proportion of individuals with low handgrip strength died during 1‐year follow‐up [23].

Further research on the oldest old is crucial for several reasons. This rapidly growing demographic presents unique challenges to healthcare systems and may have distinct physiological characteristics compared with younger elderly. The accelerated rate of strength decline in this age group [5], coupled with high prevalence of multimorbidity and frailty [2, 3], makes understanding modifiable risk factors like muscle strength critical for maintaining quality of life and independence. Moreover, previous studies primarily used categorical analyses (e.g., tertiles of muscle strength) or linear models, which may not fully capture the nature of the association between muscle strength and mortality risk in this age group. Understanding whether there are specific thresholds or a more gradual association is crucial for providing practical recommendations about target levels of muscle strength in the oldest old. Furthermore, previous studies in the oldest old were small and not representative of a broader population. Given the differences in life expectancy between men and women across different countries [24], large‐scale studies with representative samples from multiple nations are needed to investigate these associations in both sexes and provide general public health recommendations.

The aim of the present study is to determine the prospective association of handgrip muscle strength with all‐cause mortality in the oldest old (90+ years). To address the knowledge gaps of previous studies, we use a population covering 28 countries and perform restricted cubic splines analyses of the level of handgrip muscle strength with the risk of all‐cause mortality. This approach allows us to examine the potentially nonlinear relationship between handgrip muscle strength and the risk of all‐cause mortality, providing a more nuanced understanding of this association in a diverse, multinational sample of the oldest old.

## 2 Methods

### 2.1 Study Design and Participants

This study uses data from eight waves (1, 2, 4, 5, 6, 7, 8 and 9) of the Survey of Health, Ageing and Retirement in Europe (SHARE) study, which includes 28 countries (27 European countries and Israel) [25, 26]. Data were collected from February 2004 to December 2022. Wave 3 did not include data on handgrip muscle strength and was therefore discarded for the present analyses.

SHARE uses a multistage stratified sampling design in which involved countries are divided into different strata in relation to geographical area, and municipalities or zip codes within these strata served as primary sampling units [25]. Data in each SHARE wave are collected every other year through home computer‐assisted personal interviews. SHARE uses ex ante harmonized interviews, and new respondents were added in each wave to compensate for losses. The SHARE target population consists of all persons aged 50 years and over at the time of sampling who have their regular domicile in the respective SHARE country. In SHARE, persons are excluded from baseline or refreshment samples if they are incarcerated, hospitalized or out of the country during the entire survey period; are unable to speak the country's language(s); or have moved to an unknown address. In all waves, current partners living in the same household are interviewed regardless of their age.

**FIGURE 1:**
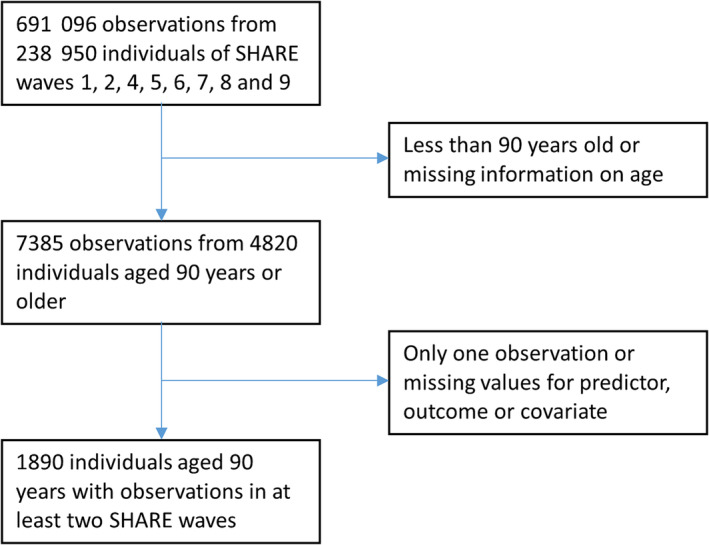
Flow of participants through the study.

The combined eight waves (1, 2, 4, 5, 6, 7, 8 and 9) contained 691 096 observations from 238 950 individuals, of which there were 7385 observations from 4820 individuals who were 90 years or older. In the present analyses, we included only individuals with observations in at least two waves of SHARE, being at least 90 years old during the first of these respective waves and not having missing values for the predictor, outcome and control variables. These criteria led to a final sample size of 1890 participants. Figure 1 shows the flow of participants through the study.

### 2.2 Predictor: Muscle Strength

Muscle strength was assessed using handgrip dynamometry (unit: kilogram). Using a handheld dynamometer (Smedley, S Dynamometer, TTM, Tokio, 0–100 kg), each hand was measured two times under the instruction of trained interviewers. Participants held their elbow at 90° flexion in a standing or sitting position, with the wrist in neutral position and upper arm vertically against the trunk. Trained interviewers provided standardized instructions to squeeze the dynamometer with maximum effort for 2 s. For the present analyses, handgrip strength was defined as the maximum value of either hand.

### 2.3 Outcome: All‐Cause Mortality

Mortality was determined from end‐of‐life interviews with proxy respondents, that is, a relative, a household member, a neighbour or any other person close to the deceased participant, who provided information on the date and cause of death. Because of the relatively small sample size of persons aged 90 years or older, we only performed the analyses with all‐cause mortality as outcome and not cause‐specific mortality. In case of a missing value for the date of decease, we set the value to the mean of the date of the last participant interview and the date of the end‐of‐life proxy interview.

### 2.4 Covariates

We controlled the analyses for sex, age, smoking status, body mass index (BMI), marital status, education, geographical region and self‐perceived health. The interviewer noted the sex of the respondent based on observation and asked the respondent in case of uncertainty. Age was calculated as date of interview minus self‐reported date of birth. Smoking status was based on two questions about whether the respondent had ever smoked and was currently smoking and was recoded into one variable (current smoker, ex‐smoker, never smoked). BMI was calculated from self‐reported weight and height of the respondent (kg·m^−2^). Respondents also replied to a question with six categories about marital status that was recoded into four categories: (1) married and living together with spouse or in a registered partnership; (2) divorced or married, but living separated from spouse; (3) never married; and (4) widowed. Education was based on the question ‘What is the highest school leaving certificate or school degree that you have obtained?’ with response categories recoded into three ISCED‐1997 categories (lower, medium and higher education) [27]. Country (28 categories) was recoded into a new variable, geographical region, with five categories (Eastern, Western, Southern and Northern Europe and Israel) according to the United Nations definition [28]. Self‐perceived health was based on the question ‘Would you say your health is…’ with five response categories: (1) excellent, (2) very good, (3) good, (4) fair, and (5) poor.

The selection of these covariates was based on their associations with both muscle strength and mortality in older adults [11]. Age and sex are fundamental demographic factors known to influence both muscle strength and mortality risk. Smoking status and BMI are important lifestyle and health indicators that can affect both muscle function and overall survival. Marital status and education serve as proxies for social support and socioeconomic status, respectively, which have been linked to health outcomes in older adults. Geographical region was included to account for potential variations in healthcare systems and environmental factors across different countries. Self‐perceived health was included as a general indicator of overall health status, which could influence both muscle strength and mortality risk.

### 2.5 Statistical Analyses

All analyses were performed in SAS version 9.4. Using the phreg procedure, we performed Cox regression to determine the prospective association of handgrip strength with mortality. Time‐on‐study in months was the timescale. We used the Breslow method for handling tied event times. To allow for potential nonlinearity and gradual associations, we used restricted cubic splines with handgrip strength as continuous measure. Based on the recommendations by Harrell [29], prespecified that knots were placed at the 10th, 50th and 90th percentiles of the exposure distribution for (1) all participants, (2) men and (3) women, respectively. To test the robustness of the results, we also performed sensitivity analyses with alternative knot placements (4 knots at 5th, 35th, 65th and 95th percentiles; 5 knots at 5th, 27.5th, 50th, 72.5th and 95th percentiles) and exclusion of those with less than 2 years follow‐up. All analyses were controlled for age, sex, smoking, BMI, marital status, education, geographical region and self‐perceived health. We used time‐varying Cox regression to account for changes in both muscle strength and covariates over time before reaching the last observation (censoring or mortality). To test for possible sex differences in the association between handgrip strength and mortality, we included a sex‐by‐handgrip strength interaction term in our Cox regression model. Likewise, we included a geographical region by handgrip strength interaction term, but this was not significant (*p* = 0.85) and was therefore left out of the final model. Analyses were performed as complete case analyses. Results are reported as hazard ratios (HRs) with 95% confidence intervals.

## 3 Results

Table 1 shows the baseline characteristics of the study population of participants aged 90 years or older. There were more women (61.6%) than men (38.4%) in the sample. Men had a higher handgrip strength than women (mean: 26.7 vs. 16.4 kg). There were only few current smokers (2.9%). The majority had a lower education (65.7%). More women (76.7%) than men (37.9%) were widowed. The majority rated their health to be fair (40.3%) or good (32.5%).

**TABLE 1:** Descriptive baseline characteristics of the study population.

	All participants				Men				Women			
	*n*	Mean	SD	Freq(%)	*n*	Mean	SD	Freq(%)	*n*	Mean	SD	Freq(%)
Handgrip strength (kg)	1890	20.4	8.0		725	26.7	7.5		1165	16.4	5.4	
Age	1890	91.0	1.5		725	90.9	1.3		1165	91.1	1.6	
Body mass index (BMI)	1890	25.0	3.9		725	25.3	3.4		1165	24.8	4.2	
Sex												
Men	725			38.4	725			100.0				
Women	1165			61.6					1165			100.0
Smoking status												
Current smoker	55			2.9	28			3.9	27			2.3
Ex‐smoker	517			27.4	361			49.8	156			13.4
Never smoked	1318			69.7	336			46.3	982			84.3
Education (ISCED‐1997)												
Lower	1241			65.7	401			55.3	840			72.1
Medium	418			22.1	179			24.7	239			20.5
Higher	231			12.2	145			20.0	86			7.4
Marital status												
Married and living together with spouse or in a registered partnership	557			29.5	411			56.7	146			12.5
Divorced or married, but living separated from spouse	74			3.9	19			2.6	55			4.7
Never married	90			4.8	20			2.8	70			6.0
Widowed	1169			61.9	275			37.9	894			76.7
Geographic region (United Nations definition)												
Eastern Europe	171			9.1	75			10.3	96			8.2
Northern Europe	454			24.0	160			22.1	294			25.2
Southern Europe	509			26.9	206			28.4	303			26.0
Western Europe	700			37.0	254			35.0	446			38.3
Israel	56			3.0	30			4.1	26			2.2
Self‐perceived health												
Excellent	60			3.2	23			3.2	37			3.2
Very good	162			8.6	72			9.9	90			7.7
Good	615			32.5	253			34.9	362			31.1
Fair	762			40.3	284			39.2	478			41.0
Poor	291			15.4	93			12.8	198			17.0

Over a mean follow‐up of 4.2 years (SD 2.4), more than half of the participants died (*n* = 971, 51.4%). Among men and women, respectively, 401 (55.3%) and 570 (48.9%) died.

**FIGURE 2:**
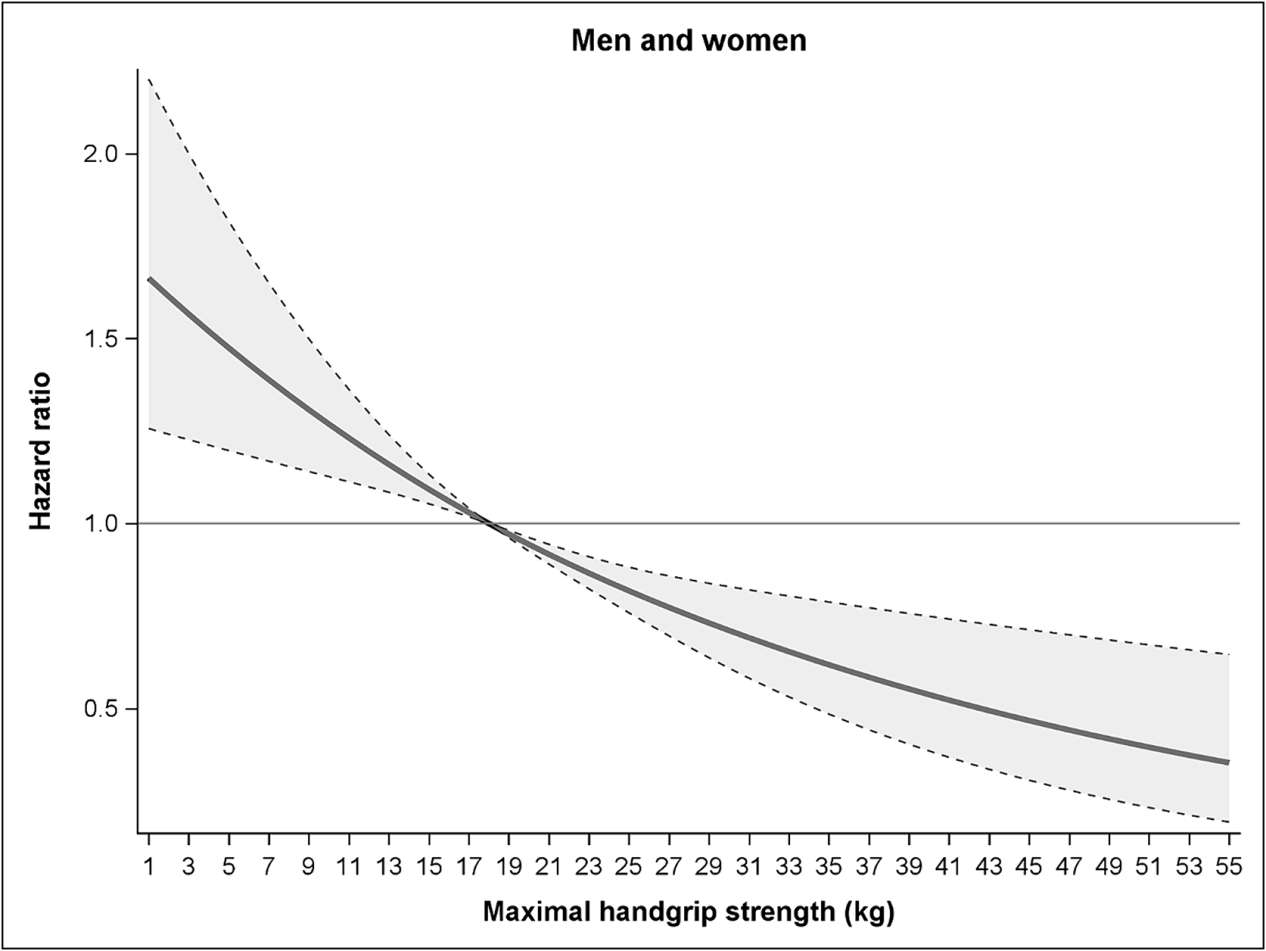
Association of handgrip strength with mortality risk during follow‐up in adults older than 90 years (*n* = 1890). The full line shows the hazard ratios and the dotted lines show the 95% CI.

Figure 2 shows the gradual association of handgrip strength with mortality risk during follow‐up in the total sample of men and women older than 90 years. Using the median level of muscle strength as reference (18 kg), lower and higher levels were associated in a curvilinear fashion with higher and lower mortality risk, respectively. The 10^th^ percentile of muscle strength (10 kg) showed a HR of 1.27 (95% CI 1.13–1.43). The 90^th^ percentile (31 kg) showed an HR of 0.69 (95% CI 0.58–0.82). Sensitivity analyses using alternative knot placements yielded consistent results, although with wider 95% confidence intervals and less smooth curves, as expected with more knots (Figures S1 and S2). Sensitivity analyses excluding the first 2 years of follow‐up also yielded consistent results (Figure S3).

**FIGURE 3:**
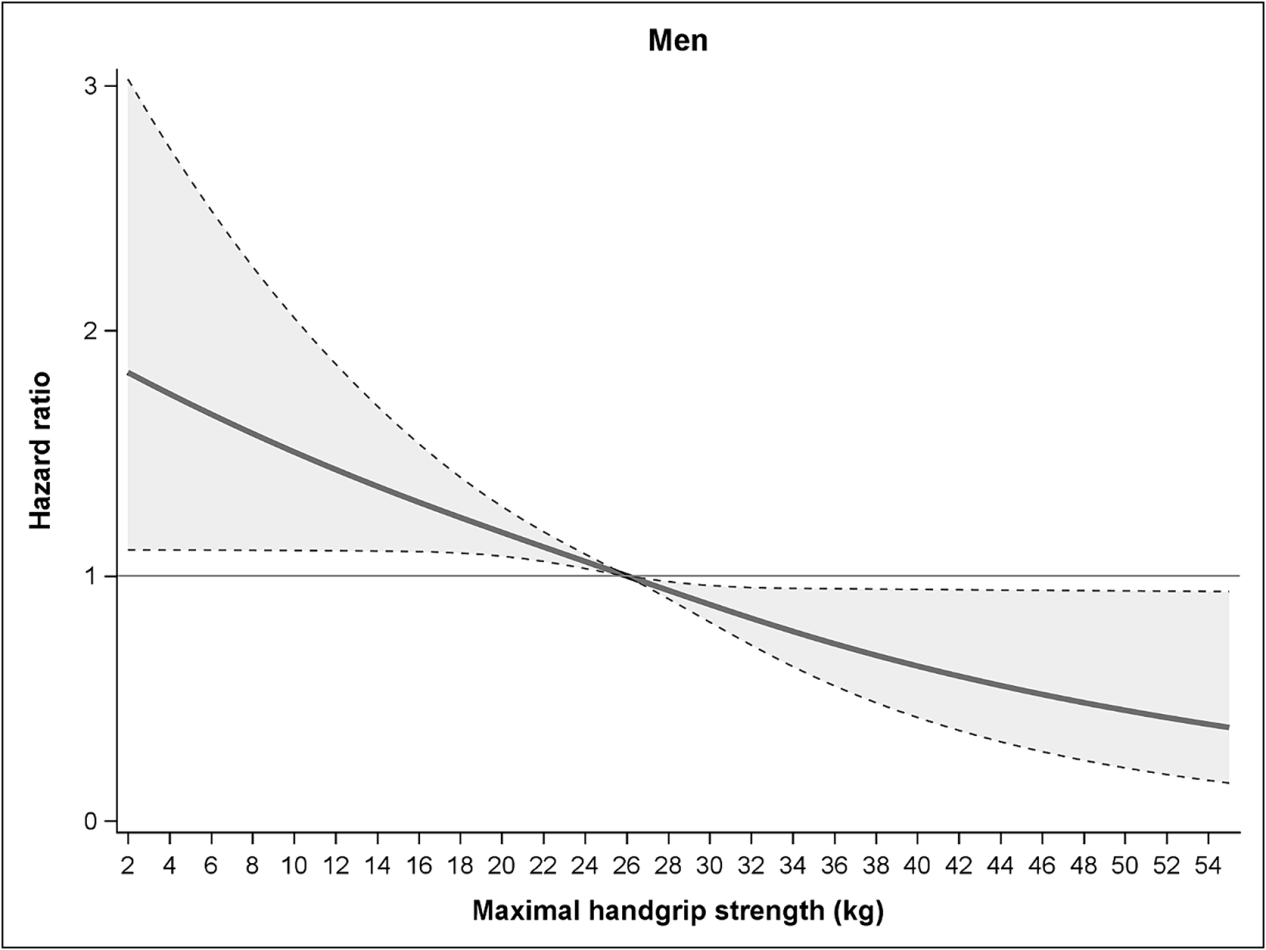
Association of handgrip strength with mortality risk during follow‐up in men older than 90 years (*n* = 725). The full line shows the hazard ratios and the dotted lines show the 95% CI.

**FIGURE 4:**
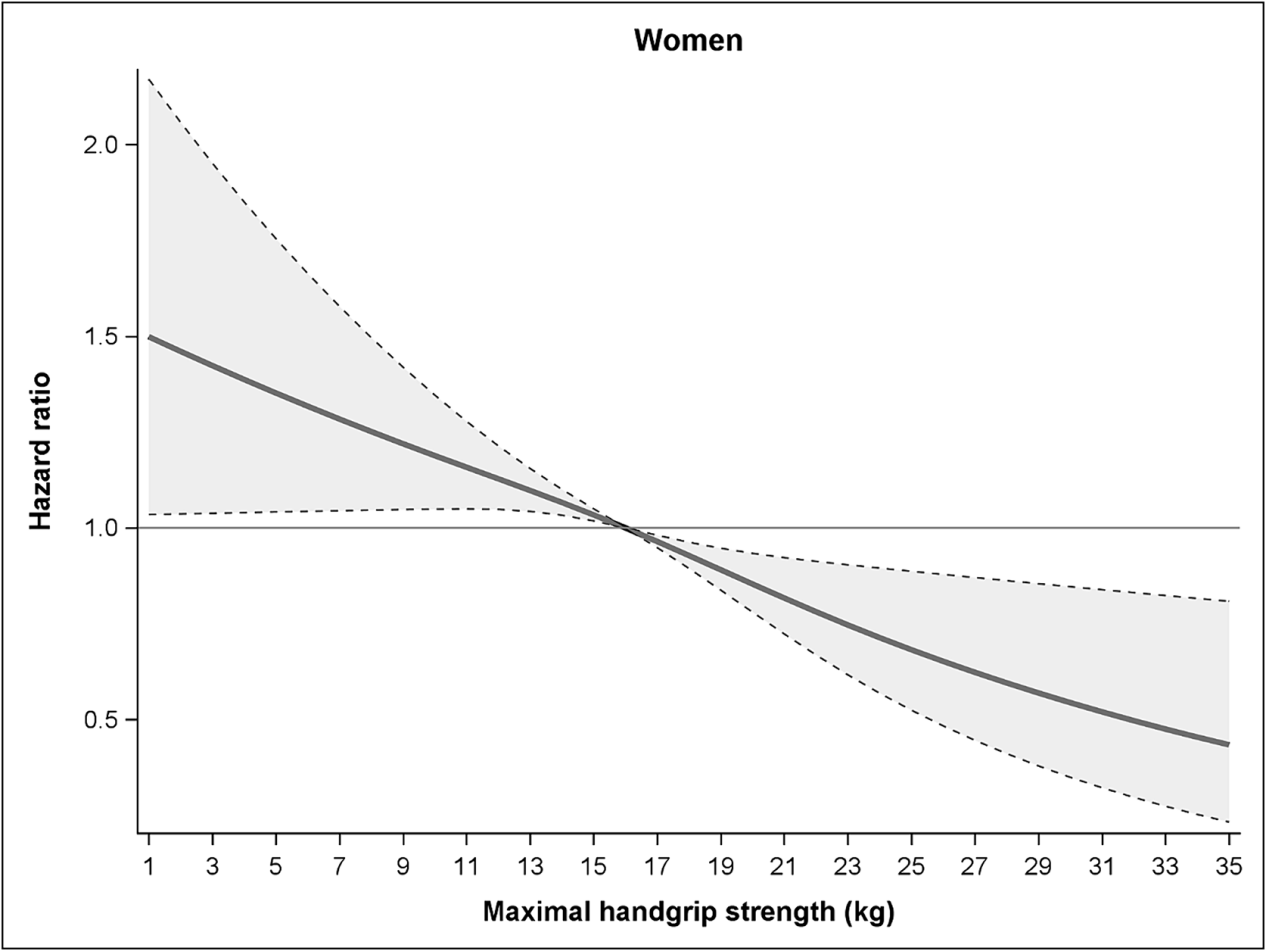
Association of handgrip strength with mortality risk during follow‐up in women older than 90 years (*n* = 1165). The full line shows the hazard ratios and the dotted lines show the 95% CI.

Sex and handgrip strength did not interact significantly (*p* = 0.40), and we there excluded the interaction term from the final model. However, as muscle strength levels differ substantially between sexes, we report the sex‐stratified analyses here. Figures 3 and 4 show the gradual association of handgrip strength with mortality risk during follow‐up in men and women, respectively, older than 90 years. Stratified for sex, the median levels of muscle strength were 26 kg for men and 16 kg for women. The 10^th^ percentile of muscle strength showed HRs of 1.33 (95% CI 1.10–1.61) at 15 kg for men and 1.19 (95% CI 1.05–1.35) at 10 kg for women. The 90^th^ percentile of muscle strength showed HRs of 0.75 (95% CI 0.59–0.95) at 35 kg for men and 0.75 (95% CI 0.62–0.90) at 23 kg for women.

## 4 Discussion

Using time‐varying Cox regression with restricted cubic splines, the present study demonstrates that muscle strength is gradually and inversely associated with mortality risk in the oldest old.

Previous studies performed in single countries have previously shown an association between handgrip strength and mortality in the oldest old, although lower age limits in these studies have varied from 80 to 90 years. For instance, research in the Netherlands [21] showed that individuals aged 85 years or older in the lowest tertile of handgrip strength had increased risk of dying during follow‐up, whereas a Belgian study [22] observed that higher handgrip strength was associated with a reduced mortality risk in those aged 80 years or older. Similarly, in Brazil, a small study involving 81 participants aged 90 or older found a higher percentage of those with a very low handgrip strength, based on the threshold for a sarcopenia diagnosis, died within a year [23]. These studies suggest the existence of a certain threshold for increased or decreased risk of mortality. However, because of the limited nature of these studies, the graded association of muscle strength with mortality risk could not be established. Importantly, the present study shows a gradual and inverse association of handgrip strength with mortality risk in the oldest old. Thus, rather than a specific threshold, every added kilogram of muscle strength appears to count.

Although handgrip strength is widely used as a proxy for overall muscle strength because of its practicality and strong correlation with other strength measures, it is important to acknowledge its limitations, particularly in the oldest old population. In individuals aged 90 years or older, handgrip strength may not fully capture the complexities of overall muscle function and its rapid changes. The ageing process can lead to differential rates of decline in various muscle groups, and handgrip strength alone may not reflect the complexity of these changes. For instance, lower limb strength, which is crucial for mobility and balance, may decline at a different rate than upper limb strength in this age group [30]. Additionally, handgrip strength does not provide information on muscle power or endurance, which are also important aspects of muscle function in older adults [31]. Future studies could benefit from incorporating multiple measures of muscle function, such as lower limb strength tests, gait speed assessments or measures of muscle power, to provide a more comprehensive picture of muscle health in the oldest old. Furthermore, given the risk for rapid declines in muscle strength at advanced ages, more frequent measurements over time would allow for a more accurate capture of the dynamic relationship between muscle strength trajectories and mortality risk. This approach could provide valuable insights into the rate of change in muscle strength and its association with mortality, rather than relying solely on absolute strength values.

The present analyses controlled for a number of potential confounders that indicate that muscle strength has an independent impact on mortality. For example, controlling for self‐perceived health excludes the possibility that the associations were simply caused by poor health in those with lower strength values. Also, the HRs continued to gradually decrease at strength levels above the median value, which again suggests that muscle strength may have an independent effect on survival probability.

We also performed sex‐stratified analyses, which is a relevant aspect of our study, because of the differences in life expectancy among men and women in European countries [24]. Although men have higher levels of muscle strength than women, a higher percentage of the men died during follow‐up in the present study. This shows the importance of supplementing the main analyses controlling for sex with sex‐stratified analyses. Using the median handgrip values as reference for each respective sex, a similar gradual pattern was observed for men and women. This shows that muscle strength in both men and women is important for survival among the oldest old.

Because of the sample size of the present population, we only used all‐cause mortality as outcome and did not investigate the specific mechanisms. However, the underlying mechanisms of our findings may be multifactorial. In the context of cardiovascular disease prevention, handgrip strength is inversely linked with markers of vascular function, including arterial stiffness [32], and increased muscular fitness has been associated with the release of cytokines and myokines into circulation, enhancing antiatherogenic properties [33]. Additionally, handgrip strength has been suggested as an indicator related to brain volumes and inflammatory states associated with dementia [34]. Studies have also highlighted the association between handgrip strength and executive functioning, indicating its potential role in cognitive impairment in older adults [35]. Furthermore, handgrip strength has been linked to learning, verbal fluency and a reduced risk of cognitive impairments such as dementia, strengthening its potential as a predictor of neurodegenerative status [36]. Thus, several underlying mechanisms may explain the present findings.

### 4.1 Practical and Clinical Implications

As muscle strength at all ages is highly adaptive to resistance training, the present findings highlight the importance of improving muscle strength in both men and women among the oldest old. This suggests that even in advanced age, interventions aimed at increasing muscle strength may potentially extend life expectancy. For example, Sahin and coworkers found in a randomized controlled trial a significant improvement in handgrip strength in institutionalized frail elderly with both low‐ and high‐intensity resistance training for 3 days a week for 8 weeks [37]. Similarly, Cadore and coworkers found in another randomized controlled trial a significant improvement in functional performance and strength outcomes after a 12‐week multicomponent exercise program consisting of resistance and power training combined with balance and gait retraining in frail nonagenarians [38]. Such type of intervention can even lead to reduced costs of care in this age group [39]. Thus, exercise intervention including resistance training has the potential to improve muscle strength and reduce cost of care in the oldest old. However, implementing strength training intervention in this vulnerable group requires careful consideration of individual health status, functional capacity and potential risks. Common concerns include cardiovascular complications, falls and musculoskeletal injuries. To minimize these risks, interventions should be personalized, progressive and supervised by trained professionals. Starting with low‐intensity exercises and gradually increasing the load as tolerated may help build strength while minimizing risks. Additionally, incorporating balance training and functional exercises can enhance overall physical performance and reduce fall risk [38]. Although challenges exist, the potential benefits of improved muscle strength on functional independence and quality of life in the oldest old likely outweigh the risks when interventions are appropriately designed and implemented.

### 4.2 Strengths and Limitations

The present study has some strengths. The use of a large, diverse population across multiple nations enhances the robustness of the present findings, suggesting that irrespective of regional and cultural differences, muscle strength serves as a key biomarker for longevity in the oldest old. The use of time‐varying Cox regression and restricted cubic splines allows for analyses of how even small changes in muscle strength may influence survival rates. The fact that the used statistical models account for changes in the status of both the exposure and the covariates strengthens the possibility of causal associations. Sensitivity analyses with different knot placements confirmed the robustness of our findings. To minimize the risk of reverse causality, we performed a sensitivity analysis excluding those with less than 2 years of follow‐up.

The present study also has some limitations. The relatively small sample size for each country and potential selection biases limits our ability to make country‐specific comparisons. Survival bias may affect our sample, as those who reach 90+ years may represent a resilient subset of the population. Several potential residual confounding factors may exist, including physical activity levels, dietary factors, access to healthcare and genetic factors. Using complete case analyses is a limitation, as participants with complete data might differ systematically from those with missing data. In addition, using proxy respondents to determine mortality status may provide some inaccuracy in death date data. On the other hand, a small study found that proxy reports showed higher agreement with adjudicated causes of death compared with death certificates, supporting the validity of this method [40]. Finally, the use of handgrip strength as a sole measure of muscle function may not fully capture the complexity of muscle health in the oldest old, and more frequent measurements could have provided insights into the rate of change in muscle strength and its association with mortality risk.

### 4.3 Conclusion

This study provides valuable evidence of a graded association between muscle strength and survival among the oldest old, highlighting the potential benefits of maintaining or enhancing muscle strength through targeted interventions, such as resistance training.

## Conflicts of Interest

The authors declare no conflicts of interest.

## Supporting information


**Figure S1** Sensitivity analysis of the results shown in Figure 2, with four (5th, 35th, 65th, and 95th percentiles) instead of three knots. Association of handgrip strength with mortality risk during follow‐up in adults older than 90 years (*n* = 1890). The full line shows the hazard ratios and the dotted lines shows the 95% CI.
**Figure S2.** Sensitivity analysis of the results shown in Figure 2, with five (5th, 27.5th, 50th, 72.5th, and 95th percentiles) instead of three knots. Association of handgrip strength with mortality risk during follow‐up in adults older than 90 years (*n* = 1890). The full line shows the hazard ratios and the dotted lines shows the 95% CI.
**Figure S3.** Sensitivity analysis of the results shown in Figure 2, with exclusion of individuals with less than two years follow‐up to avoid reverse causality. Association of handgrip strength with mortality risk during follow‐up in adults older than 90 years (*n* = 1689). The full line shows the hazard ratios and the dotted lines shows the 95% CI.

## References

[1] W. R. Frontera and J. Ochala , “Skeletal Muscle: A Brief Review of Structure and Function,” Calcified Tissue International 96 (2015): 183–195. 25294644 10.1007/s00223-014-9915-y

[2] R. S. Kamper , J. Alcazar , L. L. Andersen , et al., “Associations Between Inflammatory Markers, Body Composition, and Physical Function: The Copenhagen Sarcopenia Study,” Journal of Cachexia, Sarcopenia and Muscle 12 (2021): 1641–1652. 34708570 10.1002/jcsm.12832 PMC8718077

[3] S. Perkisas , A. De Cock , V. Verhoeven , and M. Vandewoude , “Physiological and Architectural Changes in the Ageing Muscle and Their Relation to Strength and Function in Sarcopenia,” European Geriatric Medicine 7 (2016): 201–206.

[4] E. Kennis , S. Verschueren , E. Van Roie , M. Thomis , J. Lefevre , and C. Delecluse , “Longitudinal Impact of Aging on Muscle Quality in Middle‐Aged men,” Age 36 (2014): 9689. 25104137 10.1007/s11357-014-9689-1 PMC4150881

[5] F. M. Perna , K. Coa , R. P. Troiano , et al., “Muscular Grip Strength Estimates of the U.S. Population From the National Health and Nutrition Examination Survey 2011‐2012,” Journal of Strength and Conditioning Research 30 (2016): 867–874. 26196662 10.1519/JSC.0000000000001104 PMC7197498

[6] A. Stotz , D. Hamacher , and A. Zech , “Relationship Between Muscle Strength and Gait Parameters in Healthy Older Women and Men,” International Journal of Environmental Research and Public Health 20 (2023): 5362. 37047976 10.3390/ijerph20075362 PMC10094255

[7] D. X. M. Wang , J. Yao , Y. Zirek , E. M. Reijnierse , and A. B. Maier , “Muscle Mass, Strength, and Physical Performance Predicting Activities of Daily Living: A Meta‐Analysis,” Journal of Cachexia, Sarcopenia and Muscle 11 (2020): 3–25. 31788969 10.1002/jcsm.12502 PMC7015244

[8] C. Hoffmann and C. Weigert , “Skeletal Muscle as an Endocrine Organ: The Role of Myokines in Exercise Adaptations,” Cold Spring Harbor Perspectives in Medicine 7 (2017): a029793. 28389517 10.1101/cshperspect.a029793 PMC5666622

[9] R. W. Bohannon , “Muscle Strength: Clinical and Prognostic Value of Hand‐Grip Dynamometry,” Current Opinion in Clinical Nutrition and Metabolic Care 18 (2015): 465–470. 26147527 10.1097/MCO.0000000000000202

[10] T. Rantanen , T. Harris , S. G. Leveille , et al., “Muscle Strength and Body Mass Index as Long‐Term Predictors of Mortality in Initially Healthy Men,” The Journals of Gerontology. Series a, Biological Sciences and Medical Sciences 55 (2000): M168–M173. 10795731 10.1093/gerona/55.3.m168

[11] R. López‐Bueno , L. L. Andersen , J. Calatayud , et al., “Associations of Handgrip Strength With all‐Cause and Cancer Mortality in Older Adults: A Prospective Cohort Study in 28 Countries,” Age and Ageing 51 (2022): afac117. 35639798 10.1093/ageing/afac117 PMC9351371

[12] R. López‐Bueno , L. L. Andersen , J. Calatayud , et al., “Longitudinal Association of Handgrip Strength With all‐Cause and Cardiovascular Mortality in Older Adults Using a Causal Framework,” Experimental Gerontology 168 (2022): 111951. 36096322 10.1016/j.exger.2022.111951

[13] R. López‐Bueno , L. L. Andersen , A. Koyanagi , et al., “Thresholds of Handgrip Strength for All‐Cause, Cancer, and Cardiovascular Mortality: A Systematic Review With Dose‐Response Meta‐Analysis,” Ageing Research Reviews 82 (2022): 101778. 36332759 10.1016/j.arr.2022.101778

[14] R. Mey , J. Calatayud , J. Casaña , et al., “Handgrip Strength and Respiratory Disease Mortality: Longitudinal Analyses From SHARE,” Pulmonology 30, no. 5 (2024): 445–451. 36274049 10.1016/j.pulmoe.2022.09.007

[15] R. Mey , J. Calatayud , J. Casaña , et al., “Is Handgrip Strength Associated With Parkinson's Disease? Longitudinal Study of 71 702 Older Adults,” Neurorehabilitation and Neural Repair 37 (2023): 727–733. 38116602 10.1177/15459683231207359

[16] A. Polo‐López , J. Calatayud , R. Núñez‐Cortés , L. L. Andersen , M. Moya‐Ramón , and R. López‐Bueno , “Dose‐Response Association Between Handgrip Strength and Hypertension: A Longitudinal Study of 76,503 European Older Adults,” Current Problems in Cardiology 48 (2023): 101813. 37209803 10.1016/j.cpcardiol.2023.101813

[17] E. Escourrou , F. Durrieu , B. Chicoulaa , et al., “Cognitive, Functional, Physical, and Nutritional Status of the Oldest Old Encountered in Primary Care: A Systematic Review,” BMC Family Practice 21 (2020): 58. 32220228 10.1186/s12875-020-01128-7 PMC7099824

[18] C. S. Tsoi , J. Y. Chow , K. S. Choi , et al., “Medical Characteristics of the Oldest Old: Retrospective Chart Review of Patients Aged 85+ in an Academic Primary Care Centre,” BMC Research Notes 7 (2014): 340. 24897943 10.1186/1756-0500-7-340 PMC4061508

[19] M. Kauppi , J. Raitanen , S. Stenholm , M. Aaltonen , L. Enroth , and M. Jylhä , “Predictors of Long‐Term Care Among Nonagenarians: The Vitality 90 + Study With Linked Data of the Care Registers,” Aging Clinical and Experimental Research 30 (2018): 913–919. 29222731 10.1007/s40520-017-0869-6

[20] K. Kishima , K. Yagi , K. Yamashita , et al., “Full‐Endoscopic Spine Surgery in Oldest Old Patients Aged Over 90 Years: A Case Report,” Journal of Medical Investigation 71 (2024): 169–173. 10.2152/jmi.71.169 38735715

[21] C. H. Y. Ling , D. Taekema , A. J. M. de Craen , J. Gussekloo , R. G. J. Westendorp , and A. B. Maier , “Handgrip Strength and Mortality in the Oldest old Population: The Leiden 85‐Plus Study,” CMAJ 182 (2010): 429–435. 20142372 10.1503/cmaj.091278 PMC2842834

[22] D. Legrand , B. Vaes , C. Matheï , W. Adriaensen , G. Van Pottelbergh , and J.‐M. Degryse , “Muscle Strength and Physical Performance as Predictors of Mortality, Hospitalization, and Disability in the Oldest Old,” Journal of the American Geriatrics Society 62 (2014): 1030–1038. 24802886 10.1111/jgs.12840

[23] M. L. Sáez de Asteasu , E. L. Cadore , T. Steffens , et al., “Reduced Handgrip Strength Is Associated With 1 Year‐Mortality in Brazilian Frail Nonagenarians and Centenarians,” The Journal of Frailty & Aging 13 (2024): 31–34. 38305440 10.14283/jfa.2023.21

[24] Eurostat , “Mortality and Life Expectancy Statistics,” 2022, accessed 20 May 2024, https://ec.europa.eu/eurostat/statistics‐explained/index.php?title=Mortality_and_life_expectancy_statistics.

[25] Bergmann M , Wagner M , Börsch‐Supan A . “SHARE Wave 9 Methodology: From the SHARE Corona Survey 2 to the SHARE Main Wave 9 Interview,” 2024, https://share‐eric.eu/fileadmin/user_upload/Methodology_Volumes/SHARE_Methodenband_WEB_Wave9.pdf.

[26] A. Börsch‐Supan , M. Brandt , C. Hunkler , et al., “Data Resource Profile: The Survey of Health, Ageing and Retirement in Europe (SHARE),” International Journal of Epidemiology 42 (2013): 992–1001. 23778574 10.1093/ije/dyt088 PMC3780997

[27] Eurostat , “International Standard Classification of Education (ISCED),” 2024, accessed 20 May 2024, https://ec.europa.eu/eurostat/statistics‐explained/index.php?title=International_Standard_Classification_of_Education_(ISCED).

[28] United Nations , “UNSD — Methodology”, 2024, Accessed 20 May 2024, https://unstats.un.org/unsd/methodology/m49/.

[29] F. E. Harrell , “Regression Modeling Strategies,” in With Applications to Linear Models*, Logistic and Ordinal Regression, and Survival Analysis* , 2nd ed. (Switzerland: Springer, 2015).

[30] J. Johansson , B. H. Strand , B. Morseth , L. A. Hopstock , and S. Grimsgaard , “Differences in Sarcopenia Prevalence Between Upper‐Body and Lower‐Body Based EWGSOP2 Muscle Strength Criteria: The Tromsø Study 2015–2016,” BMC Geriatrics 20 (2020): 461. 33172391 10.1186/s12877-020-01860-w PMC7654146

[31] C. Suetta , B. Haddock , J. Alcazar , et al., “The Copenhagen Sarcopenia Study: Lean Mass, Strength, Power, and Physical Function in a Danish Cohort Aged 20‐93 Years,” Journal of Cachexia, Sarcopenia and Muscle 10 (2019): 1316–1329, 10.1002/jcsm.12477. 31419087 PMC6903448

[32] D. de Lima‐Junior , B. Q. Farah , A. H. Germano‐Soares , et al., “Association Between Handgrip Strength and Vascular Function in Patients With Hypertension,” Clinical and Experimental Hypertension 41 (2019): 692–695. 30409054 10.1080/10641963.2018.1539096

[33] N. Mathur and B. K. Pedersen , “Exercise as a Mean to Control Low‐Grade Systemic Inflammation,” Mediators of Inflammation 2008 (2009): e109502. 10.1155/2008/109502 PMC2615833 19148295

[34] S. Meysami , C. A. Raji , R. M. Glatt , et al., “Handgrip Strength Is Related to Hippocampal and Lobar Brain Volumes in a Cohort of Cognitively Impaired Older Adults With Confirmed Amyloid Burden,” Journal of Alzheimer's Disease 91 (2023): 999–1006. 10.3233/JAD-220886 PMC9912728 36530088

[35] F. Herold , B. K. Labott , B. Grässler , et al., “A Link Between Handgrip Strength and Executive Functioning: A Cross‐Sectional Study in Older Adults With Mild Cognitive Impairment and Healthy Controls,” Healthcare 10 (2022): 230. 35206845 10.3390/healthcare10020230 PMC8872145

[36] K. Prokopidis , P. Giannos , T. Ispoglou , et al., “Handgrip Strength Is Associated With Learning and Verbal Fluency in Older men Without Dementia: Insights From the NHANES,” GeroScience 45 (2023): 1049–1058. 36449219 10.1007/s11357-022-00703-3 PMC9886698

[37] U. K. Sahin , N. Kirdi , E. Bozoglu , et al., “Effect of Low‐Intensity Versus High‐Intensity Resistance Training on the Functioning of the Institutionalized Frail Elderly,” International Journal of Rehabilitation Research 41 (2018): 211–217. 29620558 10.1097/MRR.0000000000000285

[38] E. L. Cadore , A. Casas‐Herrero , F. Zambom‐Ferraresi , et al., “Multicomponent Exercises Including Muscle Power Training Enhance Muscle Mass, Power Output, and Functional Outcomes in Institutionalized Frail Nonagenarians,” Age 36 (2014): 773–785. 24030238 10.1007/s11357-013-9586-z PMC4039263

[39] A. B. Bays‐Moneo , M. Izquierdo , M. M. Antón , and E. L. Cadore , “Cost‐Consequences Analysis Following Different Exercise Interventions in Institutionalized Oldest Old: A Pilot Study of a Randomized Clinical Trial,” The Journal of Nutrition, Health & Aging 27 (2023): 1091–1099.10.1007/s12603-023-2002-137997731

[40] J. H. Halanych , F. Shuaib , G. Parmar , et al., “Agreement on Cause of Death Between Proxies, Death Certificates, and Clinician Adjudicators in the Reasons for Geographic and Racial Differences in Stroke (REGARDS) Study,” American Journal of Epidemiology 173 (2011): 1319–1326. 21540327 10.1093/aje/kwr033 PMC3101067

