# Supplementary material for: Association of Muscle Strength With All‐Cause Mortality in the Oldest Old: Prospective Cohort Study From 28 Countries
Source: J Cachexia Sarcopenia Muscle. 2024 Oct 22;15(6):2756–64. doi: 10.1002/jcsm.13619 (PMC11634500; doi:10.1002/jcsm.13619)

**Supplementary Figure 1**. Sensitivity analysis of the results shown in Figure 2, with four (5th, 35th, 65th, and 95th percentiles) instead of three knots. Association of handgrip strength with mortality risk during follow-up in adults older than 90 years (n=1890). The full line shows the hazard ratios and the dotted lines shows the 95% CI.


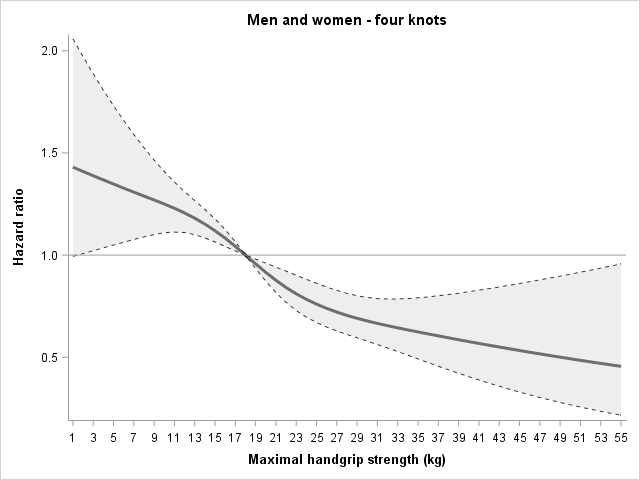


**Supplementary Figure 2**. Sensitivity analysis of the results shown in Figure 2, with five (5th, 27.5th, 50th, 72.5th, and 95th percentiles) instead of three knots. Association of handgrip strength with mortality risk during follow-up in adults older than 90 years (n=1890). The full line shows the hazard ratios and the dotted lines shows the 95% CI.


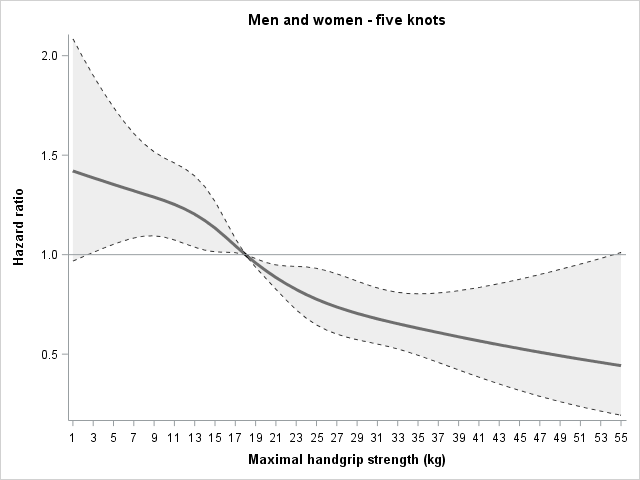


**Supplementary Figure 3**. Sensitivity analysis of the results shown in Figure 2, with exclusion of individuals with less than two years follow-up to avoid reverse causality. Association of handgrip strength with mortality risk during follow-up in adults older than 90 years (n=1689). The full line shows the hazard ratios and the dotted lines shows the 95% CI.


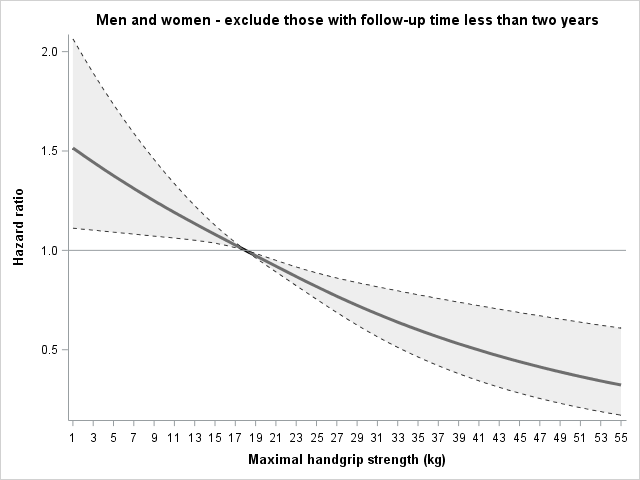

Supplement: Supplementary file 1 — Figure S1 Sensitivity analysis of the results shown in Figure 2, with four (5th, 35th, 65th, and 95th percentiles) instead of three knots. Association of handgrip strength with mortality risk during follow‐up in adults older than 90 years (n = 1890). The full line shows the hazard ratios and the dotted lines shows the 95% CI. Figure S2. Sensitivity analysis of the results shown in Figure 2, with five (5th, 27.5th, 50th, 72.5th, and 95th percentiles) instead of three knots. Association of handgrip strength with mortality risk during follow‐up in adults older than 90 years (n = 1890). The full line shows the hazard ratios and the dotted lines shows the 95% CI. Figure S3. Sensitivity analysis of the results shown in Figure 2, with exclusion of individuals with less than two years follow‐up to avoid reverse causality. Association of handgrip strength with mortality risk during follow‐up in adults older than 90 years (n = 1689). The full line shows the hazard ratios and the dotted lines shows the 95% CI. [file JCSM-15-2756-s001.docx]
